# Systemic Proteomic Analysis Reveals Distinct Exosomal Protein Profiles in Rheumatoid Arthritis

**DOI:** 10.1155/2021/9421720

**Published:** 2021-08-18

**Authors:** Qiu Qin, Ronghua Song, Peng Du, Chaoqun Gao, Qiuming Yao, Jin-an Zhang

**Affiliations:** Department of Endocrinology & Rheumatology, Shanghai University of Medicine & Health Sciences Affiliated Zhoupu Hospital, Shanghai 201318, China

## Abstract

**Objective:**

Rheumatoid arthritis (RA) is a complex disease with unknown pathogenesis. In recent years, fewer have paid attention to the broad spectrum of systemic markers of RA. The aim of this study was to identify exosomal candidate proteins in the pathogenesis of RA.

**Methods:**

Totally, 12 specimens of plasma from 6 RA patients and 6 age- and gender-matched controls from the Chinese population were obtained for nanoscale liquid chromatography coupled to tandem mass spectrometry (nano-LC-MS/MS) analysis to identify exosomal profiles.

**Results:**

A total of 278 exosomal proteins were detected. Among them, 32 proteins were significantly upregulated (FC ≥ 2.0 and *P* < 0.05) and 5 proteins were downregulated (FC ≤ 0.5 and *P* < 0.05). Bioinformatics analysis revealed that transthyretin (TTR), angiotensinogen (AGT), lipopolysaccharide-binding protein (LBP), monocyte differentiation antigen CD14 (CD14), cartilage oligomeric matrix protein (COMP), serum amyloid P (SAP/APCS), and tenascin (TNC) can interact with each other. Subsequently, these cross-linked proteins may be mainly involved in the inflammatory-related pathways to mediate the onset of RA. Noteworthy, the LBP/CD14 complex can promote the expression of IL-8 and TNF-*α*, eventually leading to the development of RA.

**Conclusions:**

Our findings suggest distinct plasmatic exosomal protein profiles in RA patients. These proteins not only take important parts in the vicious circle in the pathogenic process of RA but also serve as novel biomarkers in RA diagnosis and prognosis.

## 1. Introduction

Rheumatoid arthritis (RA) is the most common systemic autoimmune disorder affecting approximately 0.5-1% of the adult population worldwide. The disease is characterized by inflammation and proliferation of synovial tissue, destruction of cartilage and bone, and production of various autoantibodies [1] and often presents systemic manifestations including eye, skin, pulmonary, peripheral neuropathy, and cardiovascular events during progression [2]. With its prevalence constantly rising, it has become one of the main causes of disability in human and requires enormous public health resources for treatment, prevention, and sequelae amelioration.

The pathogenesis of RA involves communication between synovia cells and infiltrated immune cells such as T cell, B cell, and synovial fibroblast. The clinical picture of RA is the result of a close interaction between cells, soluble mediators, autoantibodies, and signal transduction pathways of the innate and adaptive immune system. Various studies have been conducted to find different biomarkers in disease development. Rheumatoid factor (RF) was firstly included as one of the diagnostic criteria in the 1940s [3]; then anticyclic citrullinated peptide antibody (ACPA) was discovered as an accurate diagnostic and prognostic index for RA with a higher specificity but lower sensitivity. Currently, the diagnosis of RA is mainly based on the clinical symptoms, imaging results, and some traditional laboratory tests. These methods are often used to diagnose RA during the middle-to-late disease period when treatments are unable to effectively control the disease progression [4]. Furthermore, as a complex and heterogeneous disease, about 30-40% of RA patients do not have a good response to an optimal dosing regimen of drugs including methotrexate (MTX). With the advent of biologic therapies, more prognostic markers have been used to help stratify patients for suitable treatments. In general, more efficient biomarkers need to be explored for early diagnosis and treatment of RA.

Exosomes are stable nanometer-sized (50-100 nm) membranous extracellular vesicles secreted from different type of cells to a variety of biological fluids, including blood, urine, and synovial fluids. Exosomes can deliver varieties of proteins, lipids, DNA, and RNA both locally and distantly and interact with cells to participate intercellular communication [5]. Because of their small size, stability, biologically active content, and specific targeting, exosomes, as a natural delivery system, may become a promising therapeutic modality. In addition, their carry-on contents not only reflect the physiological or pathological conditions of parental cells but also can mediate intercellular communication and functionally alter their recipient cells [6]. Unlike other conventional delivery systems, exosomes can potentially avoid degradation, go through barriers, and deliver cargo directly into the cytoplasm [7]. Moreover, exosomes secreted by brain tumor cells carry disease-specific, immunosuppressive, and carcinogenic factors [8]. Recent studies have shown that exosomes are involved in inflammatory processes of cancer, inflammatory bowel diseases, type 2 diabetes mellitus, obesity, and neurodegenerative diseases. Although few studies have revealed the abnormal expression of exosomes in RA patients, research on their function in RA still remains in its infancy. Hence, in this study, we implemented a case-control study to explore the proteomic profiling of plasma-derived exosomes in RA patients.

## 2. Materials and Methods

### 2.1. Sample Collection

From April to August 2019, blood samples were obtained from 6 RA patients and 6 gender- and age-matched healthy controls from the Outpatient Clinic of the Endocrinology and Rheumatology Department of Zhoupu Hospital (Shanghai, China). All participants were Chinese in origin, and the samples in our study needed to be at fasting state when their whole blood were drawn. Clinical and demographic data are shown in Table 1. RA patients were diagnosed according to the criteria by 2010 ACR/EULAR. None of the patients had received therapy. The controls showed negative RF, ACAP, and inflammatory indexes (such as ESR, CRP, and SAA) and are not concomitant with other inflammatory and autoimmune diseases. Whole blood samples were centrifuged at 4°C at 1500g for 10 min and at 2000 g for 20 min to separate out plasma. All plasma samples were stored at -80°C until use.

All subjects received written informed consent for participation. The study was approved by the Ethics Committee of Zhoupu Hospital.

### 2.2. Exosomal Protein Separation and Quality Control

Plasma exosomes were extracted using the magnetic bead sorting method. Briefly, 1 ml plasma was mixed with 5 ml magnetic bead (Stainless Steel Beads, Qiagen, Germany). After being washed for three times, exosomal proteins were lysed using Lysis Buffer 3 containing 1 mM phenylmethylsulfonyl fluoride (PMSF) and 2 mM ethylenediaminetetraacetic acid (EDTA). After vortexing (vortex-genie 2, Shanghai, China) and standing for 5 min, dithiothreitol (DTT) was added into the mixture to final concentration of 10 mM. The mixture was then centrifuged at 25,000g for 20 min at 4°C, and the supernatant was collected. After being incubated at 56°C for 1 h, the proteins were precipitated by adding four times volume of cold acetone and incubating at room temperature for 45 min in the dark. After centrifugation at 25,000g for 20 min, the pellets were collected and resuspended in X buffer. The concentration of plasma exosomal proteins was measured using the Bradford assay (Bio-Rad, Hercules, CA). The integrity quality control of the collected proteins was verified by protein electrophoresis on 12% uniform SDS-polyacrylamide gels using the Ettan DALT II system (Amersham Bioscience, Uppsala, Sweden) at 120 V for 120 min. The separated proteins were visualized by Coomassie blue staining.

### 2.3. Exosomal Protein Enzymolysis

Approximately 100 *μ*g of the extracted exosomal proteins were digested using trypsin (1 : 40 *w*/*w*, Promega, Madison, USA) at 37°C for 12 h. The peptides were further purified using Strata-X-C solid phase extraction system (Phenomenex, Torrance, USA), lyophilized by speed-vacuum to remove the reaction solvents, and dissolved in 20 mM NH_4_FA, pH 10 for the following experiments.

### 2.4. High pH Reversed-Phase High-Performance Liquid Chromatography (Hi-pH RP HPLC)

A total of 200 *μ*g mixed exosomal proteins were separated by high-pH RP HPLC. Basically, peptides diluted in solution A (5% ACN, pH 9.8) were injected onto a high-pH RP column (Luna C18 column with an inner diameter of 4.6 mm and a length of 250 mm, Phenomenex, CA, USA) and eluted by step linear elution program, that is, 0-10 min fast elution from 5% of solution B (95% ACN, pH 9.8), 10-40 min linear elution from 5% to 35% of solution B, and 40-41 min washing elution from 35% to 95% of solution B. The separation was performed in a Prominence HPLC system (Shimadzu, Nakagyo-ku, Kyoto, Japan) at the flow rate of 1.0 ml/min with monitoring wavelength of 214 nm and collection of one component per minute. At last, 10 fractions of peptides isolated by RP method were lyophilized.

The labeled peptides were dissolved in 0.1% formic acid (FA) solution. The supernatants were collected after centrifugation at 20,000g for 10 min. The samples were injected onto an UltiMate 3000 UHPLC (Thermo Fisher Scientific, Waltham, MA, USA) trap column (150 *μ*m i.d., C18 1.8 *μ*m x 25 cm) and eluted at a flow rate of 500 ml/min using the following gradient method: 0-5 min with mobile phase of 5% solution B (98%ACN, 0.1%FA), 5-160 min with 5%-35% solution B, 160-170 min with 35%-80% solution B, 170-175 min with 80% solution B, and 176-180 min with 5% solution B.

### 2.5. Nanoscale Liquid Chromatography Coupled to Tandem Mass Spectrometry (nano-LC-MS/MS) Analysis

#### 2.5.1. Data-Dependent Acquisition (DDA) MS Analysis

The peptides separated by liquid phase were delivered onto a nanoESI system setting in the positive ion mode. Then, the elutes were directly entered Q-Exactive HF (Thermo Fisher Scientific, San Jose, CA) to conduct DDA with primary MS scan from 350 to 1500 *m*/*z* at resolution of 60,000, and MS/MS scan at 100 *m*/*z* with resolution of 15,000. The screening conditions for the second-stage fragmented precursor were set as ion peak intensity exceeding 10,000 and top 20 precursor ions. The raw MS/MS data were converted into MGF format by Proteome Discoverer 1.2 (Thermo Fisher Scientific, Waltham, MA, USA) and searched by Mascot 2.3.02 (Matrix Science, Boston, MA, USA) against the UniprotKB/Swiss-Prot human protein database (http://www.uniprot.org).

#### 2.5.2. Data-Independent Acquisition (DIA) MS Analysis

The peptides separated by liquid phase were delivered onto a nanoESI system setting in the positive ion mode. Then, the elutes were directly entered Q-Exactive HF (Thermo Fisher Scientific, San Jose, CA) to conduct DIA with primary MS scan from 350-1500 *m*/*z* at resolution of 120,000, and fragments with 350-1500 Da were divided into 40 windows for fragmentation and signal acquisition. The DIA data were analyzed using Spectronaut software, and the bioinformatics analysis was simultaneously performed in this process.

After data processing for relative quantification of exosomal proteins, proteins with fold change (FC) > 2.0 and *P* value < 0.05 were considered significant differentially expressed proteins (DEPs).

### 2.6. Statistical Analysis

The statistical significance of differences in parameters of clinical features between the RA and controls was calculated by Student's *t*-test. The statistical significance of differences in exosomal proteins intensity between the 2 groups was calculated by a one-way analysis of variance. A *P* value < 0.05 was considered statistically significant.

## 3. Results

### 3.1. Demographic Characteristics and Clinical Features

The clinical and serological features of the 6 RA patients (2 males and 4 females) are shown in Table 1. Their median age was 55 years, ranging from 43 to 66 years. The median age of the 6 age- and gender-matched healthy controls (2 males and 4 females) was 56.5 years, ranging from 40 to 60 years. All RA patients had elevated RF and ACPA levels, and no one has received any medication.

### 3.2. The Unique Protein Profiles of Plasma-Derived Exosomes

**Figure 1:**
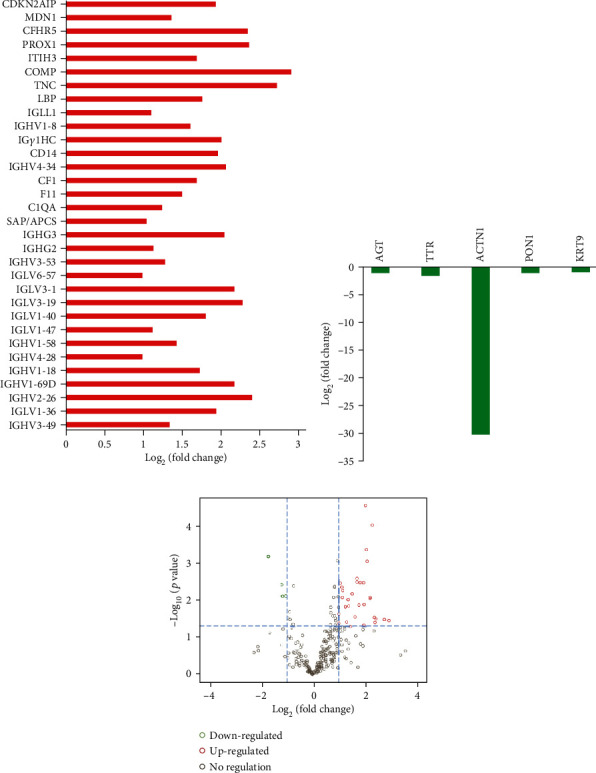
The exosomal protein profiles in rheumatoid arthritis patients. (a) The upregulated differentially expressed proteins. Details for abbreviated protein names are shown in Tables 2 and 3. (b) The downregulated differentially expressed proteins. (c) Volcano plots of differentially expressed proteins based on protein abundance changes and *t*-test (red circle: upregulated proteins; green circle: downregulated proteins; grey circle: no difference).

A total of 278 exosomal proteins were detected in all samples with high confidence. Among them, 37 were differentially expressed. In detail, 32 were upregulated and 5 were downregulated in RA patients (*P* < 0.05). Although most upregulated proteins were related to immunoglobulins (Igs), some were closely related to inflammatory or autoimmune diseases, including monocyte differentiation antigen CD14 (CD14), lipopolysaccharide-binding protein (LBP), serum amyloid P-component (SAP/APCS), tenascin (TNC), and cartilage oligomeric matrix protein (COMP). Tables 2 and 3 and Figure 1 show the detailed information on unique protein profiles in plasma exosomes of RA patients.

The subcellular localization analysis of these 37 DEPs using WoLF PSORT software (https://psort.hgc.jp/) showed that 28 were positioned at extracellular (extr), 3 located in nucleus (nucl), 2 located in mitochondria (mito) and 2 cyto_nucleus (cyto_nucl), and 1 located in plasma membrane (plas) and 1 endoplasmic reticulum (ER), respectively.

### 3.3. Gene Ontology (GO) Enrichment of DEPs

**Figure 2:**
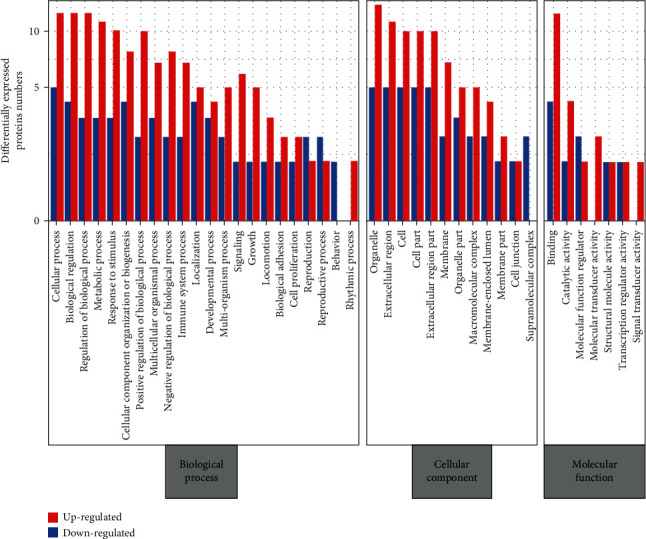
Gene ontology functional classification of plasma-derived exosomes in rheumatoid arthritis.

To obtain a global image of the proteomic changes during RA development, the DEPs were annotated with GO terms using the DAVID database (https://david.ncifcrf.gov/), based on the biological processes (BP), molecular functions (MF), and cellular component (CC). For the classification of BP, the top 10 enriched annotation terms were cellular process, biological regulation, regulation of biological process, metabolic process, response to stimulus, cellular component organization or biogenesis, positive-regulation of biological process, multicellular organismal process, negative regulation of biological process, and immune system process (Figure 2). These proteins have varied functions including binding, catalytic activity, molecular function regulator, molecular transducer activity, structural molecule activity, and signal transducer activity (Figure 2) and are cellular components of organelle, extracellular region, cell, cell part, extracellular region part, membrane, organelle part, macromolecular complex, membrane-enclosed lumen, membrane part, cell junction, and supramolecular complex (Figure 2). Impressively, from an immune-regulated point of view based on BP annotation, the significantly upregulated DEPs are enriched in the lipopolysaccharide-mediated signaling pathway and regulation of toll-like receptor signaling pathway. Besides, other inflammatory pathways that are significantly associated with DEPs, including leukocyte activation and IL-8 production, were also found uncommonly elevated in RA patients (data not shown).

### 3.4. Protein-Protein Interaction Network (PPI)

**Figure 3:**
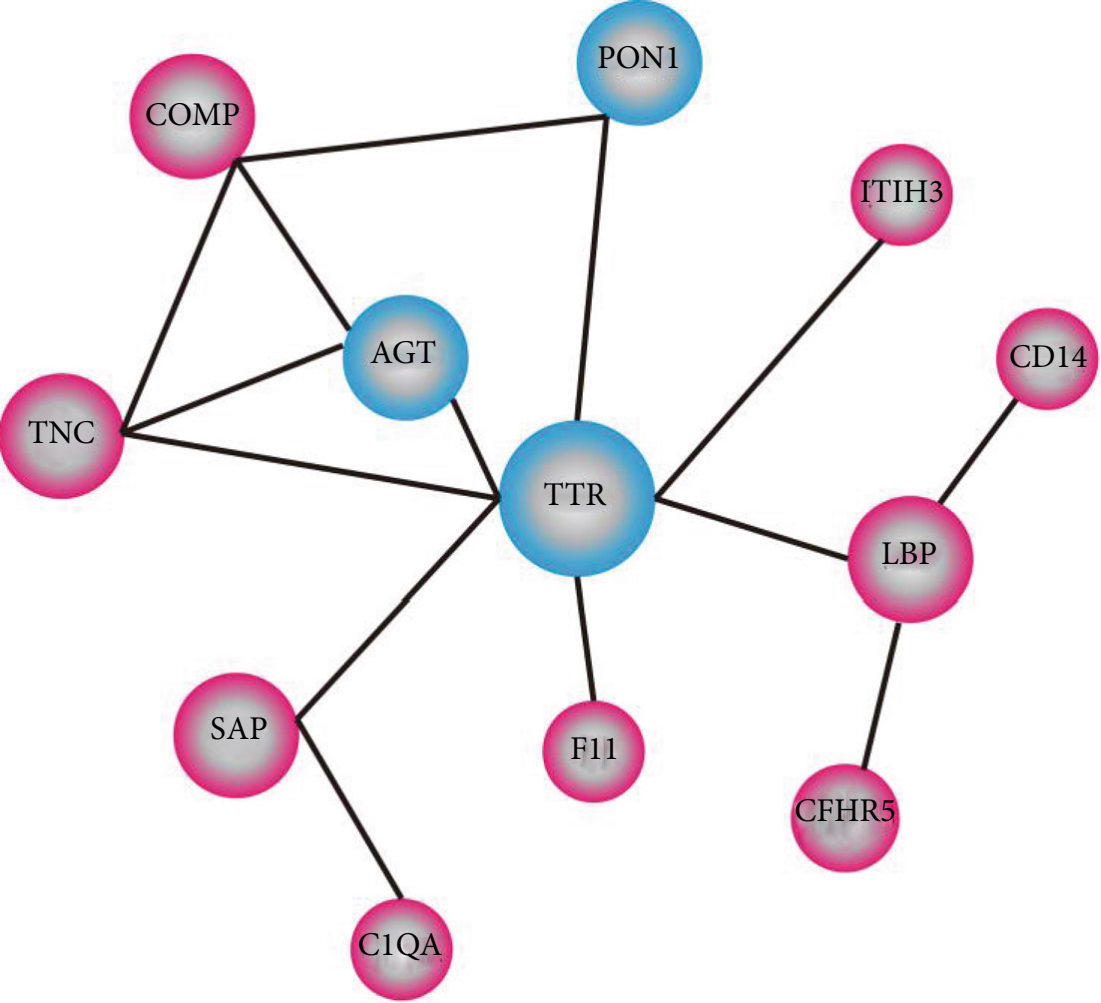
Network interaction diagram of differentially expressed exosomal proteins of rheumatoid arthritis patients compared with STRING Protein Library. ^∗^Red circle: upregulated proteins. Blue circle: downregulated proteins. The size of the circle indicates the intensity of relationship. Details for abbreviated protein names are shown in Tables 2 and 3.

Protein-protein associations of these DEPs were identified using the STRING Protein Library (https://string-db.org/), and the resulted network interaction map of the top 100 credible data is drafted and shown in Figure 3. Some unique exosomal proteins were found interacting with each other or forming regulatory networks, including LBP (P18428), CD14 (P08571), COMP (P49747), TNC (P24821), SAP (P02743), transthyretin (TTR, P02766), and angiotensinogen (AGT, P01019). These results are compatible with GO analysis, indicated that these exosomal proteins may be essential for the development of RA or have multiplicative effects on the onset of RA.

### 3.5. Pathway Analysis

**Figure 4:**
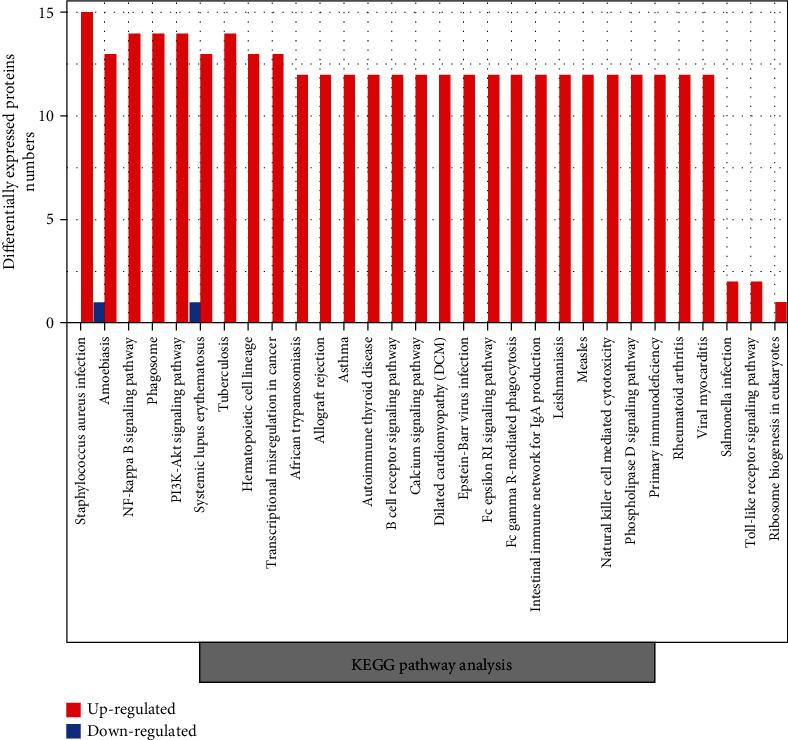
Pathway analysis of differentially expressed proteins in rheumatoid arthritis.

Pathway analysis was conducted based on the latest KEGG (Kyoto Encyclopedia of Genes and Genomes, http://www.genome.jp/kegg/) database. Almost all the DEPs upregulated in RA patients are involved in staphylococcus aureus infection, NF-kappa B signaling pathway, phagosome, PI3K/Akt signaling pathway, tuberculosis, natural killer cell-mediated cytotoxicity, B cell receptor signaling pathway, systemic lupus erythematosus (SLE), autoimmune thyroid disease (AITD), RA, and other inflammation and immune-related pathways. The five DEPs downregulated in RA proteins are involved in amoebiasis and SLE (Figure 4).

**Figure 5:**
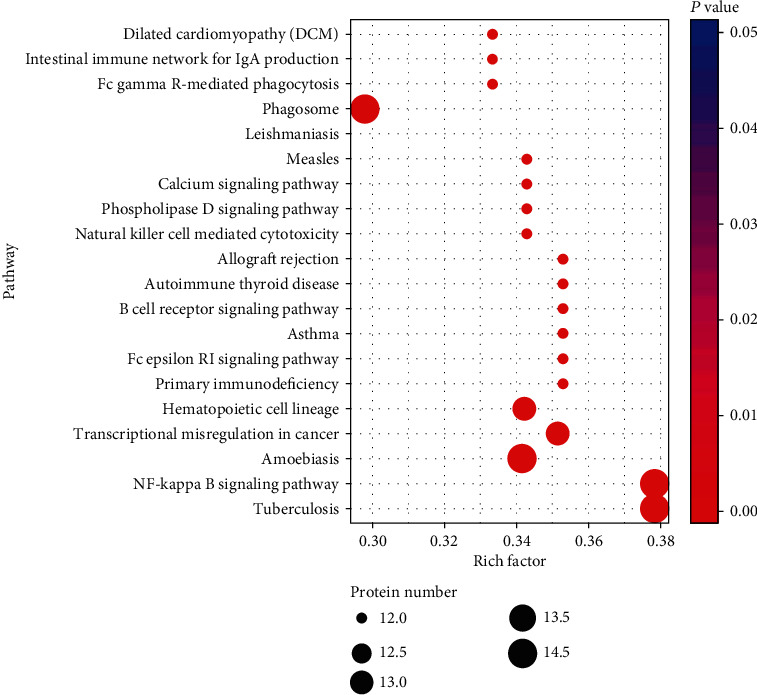
Integrated analysis based on metabolic pathways enrichment of differentially expressed proteins in rheumatoid arthritis patients compared with normal controls. ^∗^Rich factor: differentially expressed proteins annotated to pathway/all identified proteins annotated to pathway. The dot size represents the number of differentially expressed proteins annotated to pathway.

**Figure 6:**
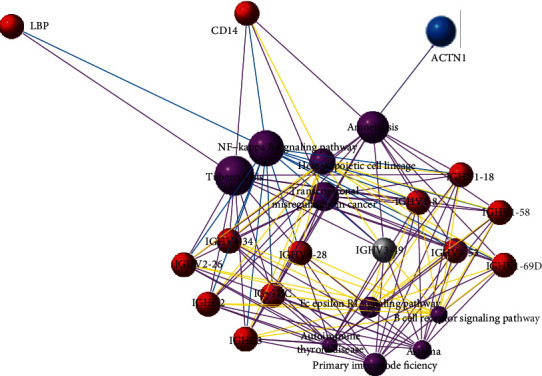
Integrate analysis based on pathways network enrichment of differentially expressed proteins in rheumatoid arthritis compared with normal controls. ^∗^Red ball: upregulated differentially expressed protein. Blue ball: downregulated differentially expressed protein. Purple ball: the top 10 enrichment pathways. Larger balls with more markers indicate more enrichment. Different colored lines represent different classification of pathways: red-cellular processes, blue-environmental information processing, green-genetic information processing, purple-human diseases, orange-metabolism, yellow-organismal systems, and brown-drug development. Details for abbreviated protein names are shown in Tables 2 and 3.

Integrated metabolic pathway analysis and pathway network relationship prompted that the top-ranking pathway with DEPs between RA patients and healthy controls was the NF-kappa B signaling pathway. Additionally, in this study, both increased LBP (P18428) and CD14 (P08571) were involved in this pathway (ko04064, details are shown in Figures 5 and 6).

## 4. Discussion

RA is a chronic disease with unknown causes. The exact entity of RA is still too complicated to be fully understood. Recently, exosomes have been attracting tremendous attention due to its powerful capability to deliver signals both intercellularly and intracellularly. As all we know, a single diagnostic biomarker is not sufficient to reflect altered body environment, especially for a heterogeneous and complex diseases such as RA. Multibiomarkers are important for the diagnosis and monitoring of these diseases [9]. Proteomics tools allow identifying global proteins and comparing protein expression patterns. In addition, proteomic analysis of protein-protein interactions and networks facilitates the identification of new proteins correlating with known proteins. In order to provide in-depth insights into the system-level molecular mechanisms of circulating exosomes in RA, the nano-LC-MS/MS method, a more sensitive proteomic technique, was used to detect plasma exosomal proteins differentially expressed between RA patients and normal controls.

We exhibited a specific circulating exosomal-protein profile with proteins enriched in inflammatory and immunoregulatory pathways. Among the total 278 exosomal proteins detected, 32 proteins were significantly upregulated and 5 were downregulated. Most of these DEPs were Igs. Further bioinformatics analysis combining GO and pathway analysis indicated that these Igs are involved in inflammatory responses of RA etiology through diverse immune regulatory pathways, such as NF-kappa B signaling pathway, Fc epsilon RI signaling pathway, primary immunodeficiency, or B cell receptor signaling pathway. Exosomes from this study as well as others showed some common signatures, such as COMP, but not exactly matched. Protein-protein interaction network analysis found some unique protein profiles in RA patients. These proteins include TTR, AGT, LBP, CD14, COMP, SAP, and TNC and can interact with each other or combine to form complexes.

Among these DEPs, COMP was the most upregulated one with already affirmed importance role in the pathogenesis of RA. COMP is primarily expressed in the cartilage and numerous other tissues/cells, including synovium, ligaments, fibroblasts, and vascular smooth muscle cells [10] and known to involve in collagen secretion and fibrillogenesis and interact with various other components of the extracellular matrix (ECM) [11]. The pathophysiological process in RA progression involves the digestion and dissolution of the intercellular components of the connective tissue by protease-derived hydrolysis. As a cartilage-derived marker of cartilage breakdown, COMP is undoubtedly considered a prognostic factor in RA, especially at an early stage. For instance, serum COMP level is significantly higher in patients with RA compared with control subjects, and its predictive accuracy is much higher than elevated ACPA level as a biomarker [12]. Moreover, both serum and synovial COMP levels are significantly correlated with the early-stage RA, especially the synovial COMP level, which increases more than twofold compared to the serum level [13]. Globally, most studies have shown that serum COMP level is higher in RA patients [14, 15]. Consistent with these studies, our data also showed elevated COMP protein level in RA patients compared to normal controls. The pathway analysis found that elevated COMP might participate in the pathogenesis of RA. Our data highlight the promising potential role of COMP as a prognostic biomarker in RA. Noteworthy is that COMP is released due to catabolic reaction and high turnover of cartilage matrix in RA. Therefore, it can be used not only for RA diagnosis but also for monitoring disease progression in patients with more advanced joint cartilage damage.

We also found upregulated levels of LBP-CD14 complex in RA patients. Previous studies have reported crosstalk between LBP and CD14. LBP extracts LPS from bacterial membranes, binds, and transfers LPS to CD14 and delivers it to the Toll-like receptor 4-myeloid differentiation 2 (TLR4-MD2) complex, which finally leads to activation of multiple signaling components and subsequent production of proinflammatory cytokines of immune cells [16]. Emerging evidences have confirmed the aforementioned proinflammatory and immune effects of LBP. For example, circulating LBP concentration is significantly higher in patients with juvenile idiopathic arthritis (JIA) than controls [17]. Plasmatic LBP and sTLR4 are associated with knee osteoarthritis progression [18]. In addition, LBP has been suggested as a new marker of synovial inflammation, and its level is significantly higher in RA patients [19, 20]. Moreover, LBP has also been proposed as a sensitive serum biomarker to evaluate RA disease activity [21]. CD14 has both conventional proinflammatory and unconventional anti-inflammatory effects in various diseases. For example, CD14 exerts significant proinflammatory impacts in the pathogenesis of metabolic diseases, such as obesity and diabetes mellitus [22]. Conversely, CD14 reduces the severity of intestinal lesions and ulcerations [23], demonstrating its protective role in autoimmune diseases. The terminal effects of CD14 in inflammation depend on many factors, such as nature of the disease, CD14 expression level, inflammation site, and competition among different CD14-dependent pathways. In line with most previous studies, we found overexpression of LBP-CD14 complex in RA patients. It is not surprising to hypothesize that increased LBP and CD14 catalyze LPS transfer to the TLR/MD2 (ko04620) complex, which can greatly activate NF-kappa B (ko04064) signaling pathway, promoting the expression of IL-8 and TNF-*α* and finally inducing the occurrence of RA.

In addition to COMP, TNC is another exosomal-protein closely related to the pathogenesis of RA. Numerous studies on arthritic animal models and RA patients have confirmed higher TNC density in RA. In murine arthritic models, TNC is arthritogenic following its intra-articular infection and its expression is required to maintain chronic joint inflammation [24]. In patients with RA, TNC level is elevated in both the RA cartilage and synovium, and soluble TNC form is detectable in synovial fluids. Additionally, high TNC is associated with more erosive joint disease and predicts poor response to biological treatment in RA patients [25]. Noteworthy, a citrullinated peptide from the fibrinogen-like globe domain of TNC is detected in RA synovial fluids, and antibodies to cyclic peptides containing citrullinated sites are found in both pre-RA and RA sera, suggesting the important role of TNC in both RA diagnosis and prediction [26]. Our data revealed TNC expression is elevated in RA patients possibly via ECM-receptor interaction pathway (ko04512) and TNC may regulate expression of miRNAs (ko05206) involved in the pathogenesis of RA. Blocking proinflammatory signals from the matrix to change the synovial microenvironment, such as TNC antagonist, may ameliorate the progression of RA without engendering global immune suppression.

SAP is another exosomal protein with multiple characteristics. SAP can bind to various molecules, then engulfed by phagocytic cells. SAP expression tends to be lowered in diverse diseases including pulmonary fibrosis, myelofibrosis, RA, and mixed connective tissue diseases, indicating that SAP deficiency might be involved in part of fibrosis [27, 28]. Few researches indicated that SAP is elevated in morbidly obese children [29], suggesting that SAP also has a proinflammatory effect. In our research, plasma SAP is enhanced in RA patients. Considering the results of other published studies, we speculate that exposure of RA-related autoantigens to immune system could lead to release chromatin, which then binds to SAP, being isolated from the antigens and subsequently driving immune responses.

Our PPI analysis also indicated that TTR and AGT participate in the protein-protein interaction network in RA patients, which is barely reported previously. TTR is increased in RA, OA, and JIA [30]. Although the mechanism underlying the enhanced TTR is not well understood, evidence has shown that TTR is one of the targets attacked by autoantibodies. AGT is reported to regulate the expression of proinflammatory transcription factor NF-kappa B [31].

Overall, we globally mapped a distinct exosomal protein profile in RA patients and indicated that these exosomes exert an immunoregulated action to trigger the development of RA. Therefore, they might be useful biomarkers complementary to RF and ACPA to, at least, partially reflect the existence and development of RA and used for RA diagnose by easily and simultaneously detecting them in a single sample with high sensitivity and specificity.

Although our sample size is sufficient to show significant differences among the clinical groups, it should be admitted that the potential application of exosomal protein profile identified in this study need to be verified in other studies with large sample size using other molecular methods.

## 5. Conclusion

In summary, our data once again demonstrated the important role of inflammatory processes in the pathogenesis of RA. Deep bioinformatics data analysis for the first time revealed the interactions among LBP, CD14, TNC, COMP, SAP, TTR, and AGT and suggested that the interaction among exosomes may play key roles in maintaining the homeostasis of the immune system-synovium axis. The complex composed of LBP and CD14 may participate NF-kappa B signaling pathway, promoting the expression of IL-8 and TNF-*α* and ultimately leading to the pathogenesis of RA. Even though further studies are needed to elucidate these points, we expect that multiexosomes founded in our study have potential application in RA diagnosis and can serve as noninvasive biomarkers.

## Figures and Tables

**Table 1:** Demographic and clinical data of patients with rheumatoid arthritis and healthy controls.

Parameter	RA patients (*n* = 6)	Normal controls (*n* = 6)	Normal range
Age (years; M ± IQR^∗^)	55 ± 13	56.5 ± 6.75	
Sex (males/females)	2/4	2/4	
Disease duration (months)	8 ± 23.5	/	
Rheumatoid factor (RF)	86.2 ± 236.03	/	0-15.9 IU/ml
Anticyclic citrullinated peptide antibody (ACPA)	148 ± 282	/	<17 U/ml
Tender joint count	3.5 ± 2.5	/	
Swollen joint count	2.5 ± 1	/	
White blood cell (WBC)	6.5 ± 3.1	6.95 ± 0.63	(3.5–9.5) × 109/l
Neutrophil (N)	3.9 ± 2.92	3.8 ± 0.9	(1.8–6.3) × 109/l
Erythrocyte sedimentation rate (ESR)	30 ± 14.5	12.5 ± 3.25	0–20 mm/h
Serum amyloid A (SAA)	15 ± 24.1	6 ± 2.5	0–10 mg/l
C-reactive protein (CRP)	7.38 ± 18.14	5.75 ± 2.75	0–10 mg/l
Prealbumin (PA)	221 ± 32.75	302.5 ± 17.75	220–400 ng/l

^∗^M ± IQR : median ± interquartile.

**Table 2:** The differentially overexpressed exosomal proteins in rheumatoid arthritis.

Protein name	Accession number	Log2 FC^∗^	*P* value
Immunoglobulin lambda variable 6-57 (IGLV6-57)	P01721	1	0.02
Immunoglobulin heavy variable 4-28 (IGHV4-28)	A0A0C4DH34	1	0.04
Serum amyloid P-component (SAP/APCS)	P02743	1.05	0
Immunoglobulin lambda-like polypeptide 1 (IGLL1)	P15814	1.12	0
Immunoglobulin lambda variable 1-47 (IGLV1-47)	P01700	1.13	0.01
Immunoglobulin heavy constant gamma 2 (IGHG2)	P01859	1.15	0.01
Complement C1q subcomponent subunit A (C1QA)	P02745	1.26	0.01
Immunoglobulin heavy variable 3-53 (IGHV3-53)	P01767	1.3	0.04
Immunoglobulin heavy variable 3-49 (IGHV3-49)	A0A0A0MS15	1.36	0.01
Midasin OS (MDN1)	Q9NU22	1.38	0.01
Immunoglobulin heavy variable 1-58 (IGHV1-58)	A0A0C4DH39	1.44	0.05
Coagulation factor XI (F11)	P03951	1.51	0.01
Immunoglobulin heavy variable 1-8 (IGHV1-8)	P0DP01	1.63	0.03
Inter-alpha-trypsin inhibitor heavy chain H3 (ITIH3)	Q06033	1.7	0
Complement factor I (CFI)	P05156	1.7	0
Immunoglobulin heavy variable 1-18 (IGHV1-18)	A0A0C4DH31	1.75	0
Lipopolysaccharide-binding protein (LBP)	P18428	1.78	0.01
Immunoglobulin lambda variable 1-40 (IGLV1-40)	P01703	1.82	0
Immunoglobulin lambda variable 1-36 (IGLV1-36)	A0A0B4J1U3	1.96	0
CDKN2A-interacting protein OS (CDKN2AIP)	Q9NXV6	1.97	0.04
Monocyte differentiation antigen CD14 (CD14)	P08571	1.97	0.01
Immunoglobulin gamma-1 heavy chain (IG*γ*1HC)	P0DOX5	2.02	0
Immunoglobulin heavy constant gamma 3 (IGHG3)	P01860	2.06	0
Immunoglobulin heavy variable 4-34 (IGHV4-34)	P06331	2.09	0
Immunoglobulin heavy variable 1-69D (IGHV1-69D)	A0A0B4J2H0	2.2	0.01
Immunoglobulin lambda variable 3-1 (IGLV3-1)	P01715	2.2	0.01
Immunoglobulin lambda variable 3-19 (IGLV3-19)	P01714	2.29	0
Complement factor H-related protein 5 (CFHR5)	Q9BXR6	2.37	0.03
Prospero homeobox protein 1 (PROX1)	Q92786	2.39	0.04
Immunoglobulin heavy variable 2-26 (IGHV2-26)	A0A0B4J1V2	2.42	0.03
Tenascin (TNC)	P24821	2.75	0.03
Cartilage oligomeric matrix protein (COMP)	P49747	2.92	0.03

^∗^FC: fold change.

**Table 3:** The differentially downregulated exosomal proteins in rheumatoid arthritis.

Protein name	Accession number	Log2 FC^∗^	*P* value
Alpha-actinin-1 (ACTN1)	P12814	-30.34	0
Transthyretin (TTR)	P02766	-1.71	0
Angiotensinogen (AGT)	P01019	-1.19	0
Serum paraoxonase/arylesterase 1 (PON1)	P27169	-1.16	0.01
Keratin, type I cytoskeletal 9 (KRT9)	P35527	-1.04	0.01

^∗^FC: fold change.

## Data Availability

The datasets used and/or analyzed during the current study are available from the corresponding author on reasonable request.
